# Severe thrombocytopaenia in patients with vivax malaria compared to falciparum malaria: a systematic review and meta-analysis

**DOI:** 10.1186/s40249-018-0392-9

**Published:** 2018-02-09

**Authors:** Cho Naing, Maxine A. Whittaker

**Affiliations:** 1 Institute for Research, Development and Innovation (IRDI), International Medical University, Kuala Lumpur, Malaysia; 2 College of Public Health, Medical and Veterinary Sciences, James Cook University, Queensland, Australia

**Keywords:** Thrombocytopaenia, Malaria, *Plasmodium vivax*, Systematic review

## Abstract

**Background:**

*Plasmodium vivax* is the most geographically widespread species among human malaria parasites. Immunopathological studies have shown that platelets are an important component of the host innate immune response against malaria infections. The objectives of this study were to quantify thrombocytopaenia in *P. vivax* malaria patients and to determine the associated risks of severe thrombocytopaenia in patients with vivax malaria compared to patients with *P. falciparum* malaria.

**Main body:**

A systematic review and meta-analysis of the available literature on thrombocytopaenia in *P. vivax* malaria patients was undertaken. Relevant studies in health-related electronic databases were identified and reviewed. The Preferred Reporting Items for Systematic Reviews and Meta-Analyses guidelines were followed. Fifty-eight observational studies (*n* = 29 664) were included in the current review. Severe thrombocytopaenia (< 50 000/mm^3^) to very severe thrombocytopaenia (< 20 000/mm^3^) was observed in 10.1% of patients with *P. vivax* infection. A meta-analysis of 11 observational studies showed an equal risk of developing severe/very severe thrombocytopaenia between the patients with *P. vivax* malaria and those with *P. falciparum* malaria (*OR*: 1.98, 95% *CI*: 0.92–4.25). This indicates that thrombocytopaenia is as equally a common manifestation in *P. vivax* and *P. falciparum* malaria patients. One study showed a higher risk of developing very severe thrombocytopaenia in children with severe *P. vivax* malaria than with severe *P. falciparum* malaria (*OR*: 2.80, 95% *CI*: 1.48–5.29). However, a pooled analysis of two studies showed an equal risk among adult severe cases (*OR*: 1.19, 95% *CI*: 0.51–2.77). This indicates that the risk of developing thrombocytopaenia in *P. vivax* malaria can vary with immune status in both children and adults. One study reported higher levels of urea and serum bilirubin in patients with *P. vivax* malaria and severe thrombocytopaenia compared with patients mild thrombocytopaenia or no thrombocytopaenia, (*P* < 0.001 in all comparisons). A pooled analysis of two other studies showed a similar proportion of bleeding episodes with thrombocytopaenia in severe *P. vivax* patients and severe *P. falciparum* patients (*P* = 0.09). This implied that both *P. vivax* and *P. falciparum* infections could present with bleeding episodes, if there had been a change in platelet counts in the infected patients. A pooled analysis of another two studies showed an equal risk of mortality with severe thrombocytopaenia in both *P. vivax* and *P. falciparum* malaria patients (*OR*: 1.16, 95% *CI*: 0.30–4.60). However, due to the low number of studies with small sample sizes within the subset of studies that provided clinically relevant information, our confidence in the estimates is limited.

**Conclusion:**

The current review has provided some evidence of the clinical relevance of severe thrombocytopaenia in *P. vivax* malaria. To substantiate these findings, there is a need for well designed, large-scale, prospective studies among patients infected with *P. vivax.* These should include patients from different countries and epidemiological settings with various age and gender groups represented.

**Electronic supplementary material:**

The online version of this article (10.1186/s40249-018-0392-9) contains supplementary material, which is available to authorized users.

## Multilingual abstracts

Please see Additional file 1 for translation of the abstract into the six official working languages of the United Nations.

## Background

*Plasmodium vivax* is the most geographically widespread species of human malaria parasites, affecting almost 40% of the world’s population [1–3]. The invisibility of the dormant liver stages of *P. vivax* to any diagnostic method may lead to an underestimation of the true prevalence of *P. vivax* far more than *P. falciparum*. This means *P. vivax* parasites could be highly prevalent in many endemic settings [3]. Most recorded *P. vivax* malaria cases originate from South East Asia and the Western Pacific, and a significant number also occur in Africa and South America [1–4]. Immunopathological studies have shown that platelets are an important component of the host innate immune responses against malaria infection [5, 6]. Thrombocytopaenia (platelet count < 150 000/mm^3^) appears to be a very frequent haematological alteration in acute malaria infections [2, 5–7]. Severe malaria is defined by a demonstration of asexual forms of the malaria parasites in the blood of a patient with a potentially fatal manifestation or complication of malaria in whom other diagnoses have been excluded [4, 8]. In brief, the clinical features of severe falciparum malaria are impaired consciousness, prostration, multiple convulsions, acute respiratory distress, circulatory collapse or shock, acute kidney injury, clinical jaundice and abnormal bleeding [8]. Although thrombocytopaenia is not included in the current World Health Organization (WHO) criteria for defining severe *P. falciparum* malaria [8], its clinical importance has been widely recognized. For instance, a retrospective analysis on 614 patients has shown that multi-organ dysfunction (MOD) and subsequent risk of death were found in patients with thrombocytopaenia [9]. However similar information on *P. vivax* malaria is limited. Since the life cycle and pathophysiology of *P. falciparum* and *P. vivax* infections are different, it is important to understand the role of platelets in *P. vivax* malaria.

There has been a recent increase in studies in the literature which have assessed the frequency of thrombocytopaenia in *P. vivax* infection. These studies varied with respect to geographical location, thrombocytopaenia grading, study design and other variables. Understanding the occurrence and role of thrombocytopaenia in *P. vivax* malaria may improve clinical practice guidelines. The aim of this study was to undertake a critical analysis of the available literature on thrombocytopaenia in *P. vivax* malaria. The overall objectives of this study were to quantify thrombocytopaenia in *P. vivax* malaria patients and to establish the associated relative risks of severe thrombocytopaenia in patients with *P. vivax* malaria compared to patients with *P. falciparum* malaria.

## Methods

The current review was done, following the Preferred Reporting Items for Systematic Reviews and Meta-Analyses (PRISMA) guideline [10] (Additional file 2).

### Literature search

One investigator searched for the original studies, addressing thrombocytopaenia in human *P. vivax* malaria. A literature search in electronic database of MEDLINE (1946 to October 2017), EMBASE (1947 to October 2017), Google Scholar, the Latin American and Caribbean Health Sciences Literature (LILACS) and African Journals Online (AJOL). The following Medical Subject Heading (MeSH) terms were used: “(vivax malaria OR vivax OR *Plasmodium vivax* OR falciparum malaria OR falciparum OR *Plasmodium falciparum*)” combined with “(platelets OR platelet counts OR thrombocytopaenia OR thrombocytopenia).” The search strategy was slightly adjusted according to the requirements of different databases. The search was limited to studies published in English until October 2017. We also checked the references of retrieved articles and relevant reviews for any additional studies.

Studies were selected if they (i) had ≥10 participants infected with *P. vivax*, residing in malaria endemic countries; (ii) were observational studies, (iii) provided data on frequency of thrombocytopaenia in *P. vivax* infection*,* compared frequency/risk of thrombocytopaenia between *P. vivax* and *P. falciparum* infection or between severe vivax malaria and uncomplicated vivax malaria/healthy controls, and (iv) provided relative risk (RR) or odds ratio (*OR*) estimates with its 95% confidence interval (*CI*) (or raw data to compute the estimates) of the association between *P. vivax* and *P. falciparum* or between severe vivax malaria and uncomplicated vivax malaria/healthy controls. If more than one study presented data from the same participants, the study with more complete information was used. Studies not meeting the above criteria were excluded. Studies assessing a special group of the population such as imported malaria or pregnant women were also excluded. This is because acquisition of immunity to malaria in people with imported malaria is different from those residing in endemic countries. Also, physiological changes in pregnant women are different from non-pregnant population.

Thrombocytopaenia was further classified into mild (100–150 000/mm^3^), moderate (50–100 000/mm^3^) [11], severe (< 50 000/mm^3^) and very severe thrombocytopaenia (< 20 000/mm^3^) [12]. The platelet count or grading used in the analysis was as reported by the authors in their published work. Parasitological diagnosis of malaria was made based on microscopy of Giemsa-stained thick/thin blood films (TBF), rapid onsite diagnostic test (RDT) or any diagnostic technique (e.g. QBC), regardless of subsequent PCR-based confirmation of the species. Severe/complicated *P. vivax* malaria and uncomplicated/non-severe *P. vivax* malaria in the current analysis were as described by the authors in the primary studies [12, 46]. The current review considered only patients with mono-infections, so patients with mixed infections were excluded.

### Data extraction and data synthesis

Two investigators individually screened the titles and abstracts, and then selected full-text articles, according to the inclusion criteria. The two investigators independently extracted information from each included study using a data extraction form prepared for this meta-analysis. This data extraction form had been pre-tested by the investigators on a sample of papers to check its utility, comprehensiveness and ease of use. Information collected was author, year of publication, country, patient characteristics (e.g. mean age, gender), study characteristics (e.g. sample size, study design, settings) and the reported clinical outcomes. Disagreements between the two investigators were resolved by consensus.

To assess the pooled proportions of thrombocytopaenia in *P. vivax* malaria, proportion data was extracted from the included studies. Raw cell counts were extracted from the studies and Freeman-Tukey double arcsine transformation and exact *CI* were employed with the individual studies for that purpose. Meta-analysis of observational studies, which assessed the risk associated with severe thrombocytopaenia in *P. vivax* malaria, was performed. The odds ratio (*OR*) of the pooled analysis was used to compare dichotomous data [13]. For the studies with mean and standard deviation (SD) of platelet counts, the mean difference (MD) and its 95% *CI* for the pooled analysis were used. When an individual study provided mean value and range, the range was transformed to SD through the formula SD = ((maximum-minimum)/4). If the studies provided median and interquartile ranges, the data was transformed into mean and SD, using a formula developed by Hozo and associates [14]. The *I*^2^ test was used for the assessment of statistical heterogeneity between studies. A value of *I*^2^ > 50% indicated substantial heterogeneity [15]. The random effects model was employed as there was substantial heterogeneity between studies. Data analysis was done by STATA 14.0 (STATACorp, Txt, USA).

## Results

**Fig. 1:**
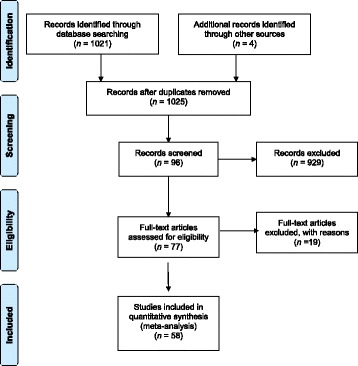
PRISMA flow chart showing the study selection process

Figure 1 provides a four-phase flow chart of the study selection process. The initial search yielded 1025 citations. After the title and abstract screening, 77 full-text papers were reviewed and a final selection of 58 studies (a total of 5536 patients had thrombocytopaenia amongst a total of 29 664 patients investigated) met the pre-specified inclusion criteria [11, 12, 16–71]. A summary of the 19 excluded studies [7, 72–89] is provided in Additional file 3.

### Characteristics of the included studies

All studies included in the present analysis used observational designs. Twenty-nine studies (50% of the included 58 studies) were prospective studies. Of them, the vast majority of participants infected with *P. vivax* (66%, 19 552/29664) were from a single large study conducted in Indonesia [11]. Half of the studies were conducted in India (50%) and the study period covered the years 1988–2015. Only 25% of the included studies had PCR confirmation of parasite species (Additional file 4).

**Fig. 2:**
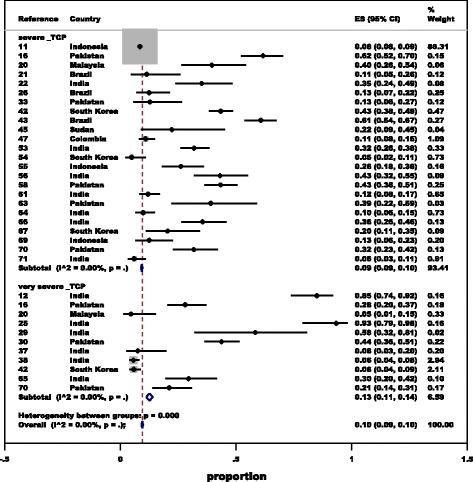
Proportion of severe to very severe thrombocytopaenia in patients with vivax malaria. TCP: Thrombocytopenia; ES: Effect estimate. The grey zone indicates a very narrow 95% upper and lower bound. The size of these grey areas reflects the weightage of their contribution to the pooled analysis. *P* value is not indicated by default, when *I*^2^ value becomes 0%

Overall, a pooled analysis showed that 18.7% (5536/29 664, 95% *CI*: 17.0–20.2%) of patients with *P. vivax* malaria had platelet counts < 150 000/mm^3^. Of these, 10.1% (2674/23 412, 95% *CI*: 9.0–10.8%) of the patients infected with *P. vivax* had either severe thrombocytopaenia (9%) or very severe thrombocytopaenia (13%) (Fig. 2). The proportions of mild and moderate thrombocytopaenia are presented in Additional file 5. There was an absence of statistical heterogeneity (*I*^2^: 0%), suggesting a high level of homogeneity between studies in each gradient of thrombocytopaenia.

**Fig. 3:**
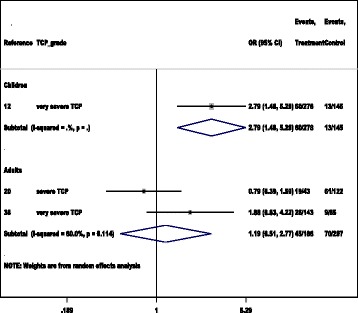
Comparison of thrombocytopaenia in patients with vivax malaria and with falciparum malaria. *OR* (95% *CI*): odds ratio and corresponding 95% confidence interval. The middle vertical line is OR 1, indicating a null value. The horizontal line indicates study specific odds ratio and corresponding 95% confidence interval. *P* value is not indicated by default, when *I*^2^ value becomes 0%. Very severe TCP denotes very severe thrombocytopenia; severe TCP denotes severe thrombocytopenia

A subgroup analysis of 11 studies (11,12,20,22,30,33,38,55,66,69,70) showed patients with *P. vivax* malaria (1930/ 20 863) and those with *P. falciparum* malaria (5895/ 38 450) had an equal risk of developing severe/very severe thrombocytopaenia, (*OR*: 1.98, 95% *CI*: 0.92–4.25) (Table 1). In a subgroup of 3 studies, a pooled analysis showed patients with severe *P. vivax* malaria and those with severe *P. falciparum* malaria had an equal risk of developing severe/very severe thrombocytopaenia [12, 20, 38]. Further stratification by age groups showed children with severe *P. vivax* malaria (60/278) had a higher risk of developing very severe thrombocytopaenia than in those with severe *P. falciparum* malaria (13/145) (*OR*: 2.80, 95% *CI*: 1.48–5.29) [12]. However an equal risk was observed for adult severe cases with *P. vivax* malaria and those with *P. falciparum* malaria (45/186 vs 70/207, *OR*: 1.19, 95% *CI*: 0.51–2.77, *P* = 0.22) [20, 38] (Fig. 3).

**Table 1:** Distribution of thrombocytopaenia between vivax malaria and falciparum malaria

Study [Reference]	thrombocytopaenia grade	Vivax malaria		Falciparum malaria		Odds ratio	95% CI
		event	total	event	total		
Taylor, 2008 [69]	severe	8	64	5	98	2.65	0.83–8.52
Poespoprodjo, 2009 [55]	severe	23	88	5	51	3.25	1.15–9.19
Khan, 2012 [33]	severe	5	39	22	82	0.4	0.14–1.15
Dhungat, 2013 [22]	severe	19	54	13	28	0.62	0.24–1.58
Barber, 2013 [20]	severe	19	43	61	122	0.79	0.39–1.59
Singh, 2014 [66]	severe	32	90	32	90	1	0.54–1.84
Haroon, 2013 [30]	severe	70	110	2	472	411.2	97.2–1739.5
Lampah, 2015 [11]	severe	1650	19 552	5722	36 759	0.49	0.47–0.52
Tanwar, 2012 [12]	very severe	60	278	13	145	2.79	1.47–5.28
Kochar, 2010b [38]	very severe	26	460	9	525	3.43	1.59–7.4
Uttra, 2010 [70]	very severe	18	85	11	78	1.64	0.72–3.72
Pooled, Odds Ratio	*I*^2^ = 94.1%					1.98	0.92– 4.25

A subset of 4 studies [24, 35, 41, 58] reported the mean platelet counts. Decreased platelet counts were recorded in patients with *P. vivax* malaria compared to the healthy controls (*P* < 0.001 in all four studies) (Additional file 6). In another subgroup of 2 studies with PCR confirmed malaria [12, 29], the mean platelet counts were almost the same in both uncomplicated *P. vivax* malaria and uncomplicated *P. falciparum* malaria (MD: -24.5, 95% *CI*: -57.8–8.6, *P* = 0.15). However, one study on severe malaria [46] showed a relatively lower mean platelet count in patients with severe *P. falciparum* malaria than in those with severe *P. vivax* malaria (MD: 46.0, 95% *CI*: 37.5–54.5, *P* < 0.0001). This implied that the platelet counts could vary with severity of malaria.

A pooled analysis of another 4 studies [12, 32, 59, 63] showed that 15% (95% *CI*: 9–21%) of *P. vivax* malaria patients with thrombocytopaenia developed minor bleeding episodes (Additional file 7). These bleeding manifestations were epistaxis, haematemesis, petechiae and purpura. A pooled analysis of 2 studies [12, 37] showed a similar risk of developing bleeding episodes for both thrombocytopaenia with severe *P. vivax* malaria (15.7%, 70/445) and with *P. falciparum* malaria (11.2%, 30/269) (*OR*: 0.67, 95% *CI*: 0.43–1.06, *P* = 0.09). This implied that both *P. vivax* and *P. falciparum* infections could present with bleeding episodes should there be a change in platelet counts in the infected patients.

### Associated risks of thrombocytopaenia in *P. vivax* malaria

Three studies with PCR confirmed *P. vivax* malaria reported parasite density. Of these, one study showed a higher parasite density among patients with severe thrombocytopaenia in *P. vivax* malaria than in those with either mild thrombocytopaenia or no thrombocytopaenia (*P* = 0.524) [26]. The remaining 2 studies [21, 38] qualitatively reported almost the same *P. vivax* parasite density in patients with thrombocytopaenia and those without thrombocytopaenia. This was indirectly supported by another study with 456 non-PCR confirmed *P. vivax,* showing no statistically significant relationship between parasite density and thrombocytopaenia (*R*^2^ = 0). This implied that parasite load was not a predictor of change in platelet counts in these patients [39].

One study showed a significantly lower haemoglobin (Hb) level with severe thrombocytopaenia (11.66 ± 2.19, *n* = 79) compared to those with moderate thrombocytopaenia (12.65 ± 1.63, *n* = 93) (*P =* 0.002) or the healthy (non-malaria) participants (12.29 ± 1.99, *n* = 100) (*P =* 0.05) [58]. This indicated that as the platelet count decreased, so did the Hb level in patients with *P. vivax* malaria. However, a small study reported that there was no significant difference in Hb levels between *P. vivax* malaria with severe thrombocytopaenia (*n* = 10) and those with mild thrombocytopaenia (*n* = 45) (13.00 ± 3.25 vs 12.90 ± 3.25) (*P =* 0.9) or no thrombocytopaenia (normal platelet counts) (*n* = 35) (13.00 ± 3.25 vs 12.10 ± 3.25) (*P =* 0.4) [26]. The same study on patients with *P. vivax* malaria reported that there were higher urea levels in patients with severe thrombocytopaenia (*n* = 10) compared to patients with mild thrombocytopaenia (*n* = 45) (41.50 ± 10.37 vs 30.00 ± 7.20) (*P* < 0.001) or no thrombocytopaenia (*n* = 35) (41.50 ± 10.37 vs 28.00 ± 7.00) (*P* < 0.001) [26]. Also, there was higher serum bilirubin levels in patients with severe thrombocytopaenia (*n* = 10) (1.70 ± 0.42) compared to those with mild thrombocytopaenia (*n* = 45) (1.30 ± 0.32) (*P =* 0.001) or no thrombocytopaenia (*n* = 35) (0.90 ± 0.25) (*P* < 0.001) [26]. Therefore, a decreasing platelet count was related to both elevated urea and serum bilirubin levels in *P. vivax* infection. A study on 51 children in India had reported that the association of severe *P. vivax* malaria in children with severe thrombocytopaenia was 2.6 times significantly greater than those with non-severe thrombocytopaenia (*OR*: 2.60, 95% *CI*: 1.20–5.52, *P* ≤ 0.014) [12].

One study has reported that anaemia with thrombocytopaenia was more common in patients with *P. falciparum* malaria (17.6%, 13/74 vs 39/103) compared to those with *P. vivax* malaria (37.9%, 39/103) (*P =* 0.004) [40].

### Mortality risk

A pooled analysis of 2 studies with PCR confirmed parasite species [40, 64] showed an equal risk of mortality with severe thrombocytopaenia in *P. vivax* malaria patients (10.2%, 5/49) and *P. falciparum* malaria patients (14%,8/57) (*OR*: 1.16, 95% *CI*: 0.30–4.60, *P* = 0.87) (Additional file 8). Another study reported a 9-fold increase in the risk of death from *P. vivax* malaria in children with severe anaemia and severe thrombocytopaenia, compared to those without anaemia or thrombocytopaenia (*OR*: 9.21, 95% *CI*: 4.53–18.73) [11].

## Discussion

Based on available data from 58 individual observational studies across 12 endemic countries, the current study has provided some evidence of the increasing risks of developing thrombocytopaenia in *P. vivax* malaria patients. The observational design in these studies was appropriate as it quantified the frequency and associated risks in regular clinical practice, reflecting real life situations. The findings suggest that thrombocytopaenia is a common manifestation equally observed in both human *P. vivax* and *P. falciparum* infections. A review of studies restricted to Brazil reported that the mortality rate in *P. vivax* malaria with severe thrombocytopaenia alone was comparable to that of *P. falciparum* malaria [4]. Our findings also suggest that both the magnitude of severe thrombocytopaenia and mortality risks are similar in both *P. falciparum* and *P. vivax* malaria. Dating back to the era of induced malaria therapy, the fatality rate with the Madagascar strain of *P. vivax* was 10–15% when used to treat patients with neurosyphilis in the United Kingdom [90]. Details of this evidence are available elsewhere [80].

Although the mechanism of thrombocytopaenia in malaria is not fully understood, numerous studies have hypothesied possible patho-immunological mechanisms of thrombocytopaenia in malaria. Oxidative stress [23], macrophage colony stimulating factor [91], immunoglobulin G binding to platelet-bound malaria antigens [73], spleen pooling [92], increased plasma cell free circulating nucleic acids levels in *P. vivax* [93] and platelet phagocytosis [21] are possible mechanisms resulting in damage to thrombocytes or the excessive removal of platelets. A case study in India reported haemophagocytosis occurring in a *P. vivax* malaria patient with a platelet count 16 000/mm^3^ [87]. Moreover, an interaction between *Plasmodium* and platelets has been postulated as *P. vivax* has been seen within the platelets of patients when using electron microscopy [94]. It is probable that thrombocytopaenia in malaria is actually a multifactorial phenomenon [21].

In our review, we found that 10.1% of *P. vivax* infected patients had severe to very severe thrombocytopaenia, regardless of confirmatory tests used for *P. vivax* malaria. Of note, a large-scale study in Indonesia [11] had contributed to this pooled proportion, and there the prevalence was 8.4% (1650/19 552). An earlier review reported that the pooled proportion of severe thrombocytopaenia was 7.5% (95% *CI*: 4.2–10.8%) amongst outpatients with *P. vivax* malaria [95]. This difference might be due to the periods covered by the included studies, variation in selection criteria used by the reviews and/or levels of malaria endemicity in the study areas. Moreover, the proportion of thrombocytopaenia will be higher in studies performed at tertiary-referral hospitals (with a higher proportion of patients with severe vivax malaria), and lower in community-based studies. In our analysis, we included published studies between 1988 and 2015. We did not include studies with imported malaria cases [72, 79, 89] nor those with pregnant women included [76, 78]. The difference in the proportion of severe thrombocytopaenia between the current review and earlier review might also be due to the drug resistance status of parasites in the study areas in the included studies. It has been found that drug resistant parasites prolong their stay in their hosts and do more damage, resulting in thrombocytopaenia [96].

### Clinical relevance

A limited number of studies included in this review documented that low Hb, and high serum bilirubin and urea levels in *P. vivax* malaria were associated with severe thrombocytopaenia. These findings implied that severe anaemia, and impaired liver and renal functions would be expected in patients with severe thrombocytopaenia and *P. vivax* malaria. This also indicates that MOD may occur in malaria patients with thrombocytopaenia. This is a similar phenomenon to that seen in *P. falciparum* infections. This provides further evidence to refute the long-held view that *P. vivax* infection is benign [5, 11, 38, 39, 80]. This misconception and may have led to inappropriate clinical management, research and control [63] of *P. vivax* malaria. This collective evidence supports the need for increased efforts to actively control and then eliminate both *P. vivax* [38] and *P. falciparum* infections.

This review reported minor bleeding episodes. Thrombocytopaenia, even in severe cases, was not commonly accompanied with severe bleeding [11, 24] or clinical bleeding in malaria [12]. This might be due to the good tolerance of a low platelet count in malaria patients through an enhanced aggregability pathway [49]. Another possibility is that larger platelets observed in thrombocytopaenic patients may compensate for the low number of platelets in the periphery, thereby preserving primary haemostasis and avoiding severe bleeding [24]. The high proportion of severe thrombocytopaenia in *P. vivax* malaria patients found in this review highlights the clinical relevance of this parameter in diagnosis and treatment. However, in the absence of (severe) bleeding manifestations in malaria patients, thrombocytopaenia alone is not regarded as a WHO criterion used to define severe malaria [8]. Additionally, other severe manifestations in the form of impaired renal and liver function, leading to MOD and increased mortality have been documented in studies from India [39] and Brazil [11] and further highlight the need for a broadened clinical awareness of *P. vivax* malaria and its complications. Although it was not included in the current analysis, a case study in India had reported retinal haemorrhage in a patient of *P. vivax* malaria who had a platelet count of 40 000/mm^3^ [86].

### Study limitations

The present study has several limitations. Not all studies included in this review used the same methods for platelet ‘labelling’. Hence, misclassification bias could potentially affect the interpretation of the results. For instance, the haemolysis necessary to count platelets in the chamber creates pseudo-platelets [97], resulting in erroneously high numbers of platelets being counted. This shortfall is avoided if an electronic counter is used in which platelets are counted in platelet rich, red blood cell free, and parasite-free plasma [72]. Moreover, semi-immune malaria patients were likely to have low parasite counts and may not have an increase in platelet count even after treatment [72].

We did not undertake segregated analysis of thrombocytopaenia by use or not of PCR confirmed speciation. This is because microscopy by technicians at the malaria clinics in the endemic countries may contribute to the relatively minor possible errors. Moreover, PCR in the studies included in the current review might have excluded errors made by microscopists in the same studies. Although the quality of the studies included was variable if *P. vivax* malaria was not confirmed by PCR, the current review attempted to minimize this problem by including studies which focused on specific groups in endemic areas where *P. vivax* is prevalent. This reinforces our confidence in the effect estimates. Nevertheless, the current work focused exclusively on thrombocytopaenia and also reported comparisons between *P. vivax* and *P. falciparum* malaria other than solely giving frequencies of each disease thereby yielding more reliable and comprehensive information.

We were unable to rule out association of co-infections such as intestinal parasitic infections [58], acute parvovirus B19 infection [98] or an endemic viral infection such as dengue in Brazil [4]. These comorbid infections in patients included in the primary studies could contribute to a substantial percentage of thrombocytopaenia [4]. One published study highlighted that acute or chronic co-morbidities were detected in 58% (14/24) of the patients with *P. vivax* in the Western Brazilian Amazon [99]. Another confounding factor may occur if patients in the primary studies had taken primaquine-containing antimalarial treatment, as they might have recovered normal platelets counts. Hence, the interpretation of the current findings needs to consider potential effects of co-infections and antimalarial medication as confounding factors.

The manifestations of thrombocytopaenia could also be influenced by context-dependent factors such as the level of malaria endemicity, accessibility to effective malaria treatment and the age-specific and gender-specific host immunity. We have found that a variation in the risk of developing thrombocytopaenia in *P. vivax* malaria between children and adults might be due to a difference in immune status between the age groups. For instance, young children exhibit an “antidisease immunity” that affects the risk and extent of morbidity associated with a given parasite density [100]. Inadequate data in the included studies preclude the ability to perform stratified analyses based on any of these potential influencing factors.

### Implications

Although further substantiation is required, one study reported that patients with mild thrombocytopaenia were about 32 times more likely to have malaria infection than those with normal platelet count (*OR*: 31.8, 95% *CI*: 25.1–39.7, *P* ≤ 0.001) [41]. An estimation of platelet counts is readily available with an automated blood counter even in some remote health posts, where other biochemical and inflammatory markers are not available. The presence of thrombocytopaenia in people from malaria endemic areas may also be useful to support the diagnosis of malaria in cases with low levels of parasite biomass [41], as it is common in natural *P. vivax* infections. Since severe thrombocytopaenia may be associated with several infectious diseases such as dengue and leptospirosis, patients with persistent thrombocytopaenia in malaria endemic areas should also be thoroughly investigated for co-infections or other identifiable causes of thrombocytopaenia.

The association of severe malaria was found to be stronger with severe thrombocytopaenia in children with a specificity of 88.3% and a positive predictive value of 85% [12]. Therefore, the presence of severe thrombocytopaenia should serve as a warning sign of poor outcome, particularly when coexisting with severe anaemia [11]. Any patient with an acute febrile illness without localized signs and having a combination of anaemia and thrombocytopaenia should raise the possibility of malaria infection, and they should then proceed to use confirmatory tests [12]. The increased mortality risk in vivax malaria cases with severe thrombocytopaenia as well as severe anaemia highlights the need for healthcare personnel to promptly examine for these signs in malaria patients living in endemic areas.

Based on the findings, patients with severe thrombocytopaenia in malaria endemic settings and without other definitive causes for the thrombocytopaenia should be treated as severe malaria [12] and the presence of severe thrombocytopaenia should be included as a defining criterion of malaria severity for both *P. falciparum* and *P. vivax* malaria [11]. The platelet count (indicating the presence of thrombocytopaenia) coupled with other clinical and microscopy parameters can significantly improve malaria diagnosis, provide timely treatment for malaria infections [41] and address the associated higher morbidity and mortality risks.

## Conclusion

The current review has provided some evidence of the clinical relevance of severe thrombocytopaenia in *P. vivax* malaria. To substantiate these findings, there is a need for future well designed, large-scale, prospective studies among patients infected with *P. vivax* from different countries and epidemiological settings with various age and gender groups represented.

## Additional files

Additional file 1: Multilingual abstracts in the six official working languages of the United Nations (PDF 739 kb)

Additional file 2: PRISMA checklist (DOC 58 kb)

Additional file 3: The excluded studies (DOC 37 kb)

Additional file 4: Characteristics of the included studies (DOC 119 kb)

Additional file 5: Proportions of mild and moderate thrombocytopaenia in vivax malaria (DOC 62 kb)

Additional file 6: Distribution of mean platelet counts (DOC 29 kb)

Additional file 7: Proportion of bleeding manifestation in patients with vivax malaria (DOC 32 kb)

Additional file 8: Comparison of mortality with severe thrombocytopaenia between severe vivax malaria and severe falciparum malaria (PDF 99 kb)

## Abbreviations

AJOL: African Journals Online
Hb: Hemoglobin
LILACS: Latin American and Caribbean Health Sciences Literature
MD: Mean difference
MOD: Multi-organ dysfunction
OR: Odds ratio
*P. falciparum*: *Plasmodium falciparum*
*P. vivax*: *Plasmodium vivax*
RDT: Rapid onsite diagnostic test
RR: Risk ratio
SD: Standard deviation
TBF: Thick/thin blood film
