# Supplementary material for: Severe thrombocytopaenia in patients with vivax malaria compared to falciparum malaria: a systematic review and meta-analysis
Source: Infect Dis Poverty. 2018 Feb 9;7:10. doi: 10.1186/s40249-018-0392-9 (PMC5808388; doi:10.1186/s40249-018-0392-9)
Supplement: Supplementary file 1 — Multilingual abstracts in the six official working languages of the United Nations (PDF 739 kb) [file 40249_2018_392_MOESM1_ESM.pdf]

Translation of the abstract into the six official working languages of the United Nations

النقص الحاد في الصفائح الدموية لدى مرضى ملاريا المتصورة النشيطة في مقابل الملاريا المنجلية: مراجعة منهجية وتحليل تجميعي

تشو نايىغ، ماكسين أيه ويتاكر

الملخص

الخلفية: المتصورة النشيطة هي أكثر أنواع الطفيليات المسببة للملاريا انتشاراً بين البشر من الناحية الجغرافية. وقد أظهرت الدراسات الباثولوجية المناعية أن الصفائح الدموية مكون مهم من مكونات الاستجابة المناعية الفطرية لدى المضيف ضد عدوى الملاريا. من ثم، تمثلت أهداف هذه الدراسة في تحديد مدى النقص في الصفائح الدموية لدى مرضى ملاريا المتصورة النشيطة، إلى جانب تحديد المخاطر المحتملة المرتبطة بالنقص الحاد في الصفائح الدموية لدى مرضى ملاريا المتصورة النشيطة في مقابل الملاريا المنجلية.

مجموعة الأبحاث الرئيسية: أجرينا مراجعة منهجية وتحليلاً تجميعياً للدراسات السابقة المتاحة حول نقص الصفائح الدموية في حالات ملاريا المتصورة النشيطة. كما أجرينا بحثاً عن الدراسات ذات الصلة في قواعد البيانات الإلكترونية المتعلقة بمجال الصحة، متبعين في ذلك *المبادئ التوجيهية لعناصر التقارير المفضلة للمراجعات المنهجية والتحليلات* التجميعية تضمنت المراجعة القائمة ثمانٍ وخمسين دراسة رصدية (حجم العينة = 29,664). وقد لوحظ وجود نقص في الصفائح الدموية يتراوح بين الحاد (<50,000/ ملم ^{3}) والشديد (<20,000/ ملم ^{3}) في 10.1% من المرضى المصابين بعدوى المتصورة النشيطة. كذلك، فقد أظهر التحليل التجميعي لـ11 دراسة رصدية وجود احتمالية مماثلة للإصابة بالنقص الحاد/ الحاد الشديد في الصفائح الدموية بين مرضى ملاريا المتصورة النشيطة والملاريا المنجلية (نسبة الأرجحية : 1.98, 95% مجال الثقة: 0.92-4.25). مما يوضح أن نقص الصفائح الدموية سمة شائعة من سمات ملاريا المتصورة النشيطة والملاريا المنجلية على حد السواء. كذلك، فقد بينت إحدى الدراسات ارتفاع احتمالية الإصابة بالنقص الحاد في الصفائح الدموية لدى الأطفال المصابين بملاريا المتصورة النشيطة الحادة عن المصابين بالملاريا المنجلية الحادة (نسبة الأرجحية: : 2.8, 95% مجال الثقة: : 1.48-5.29). غير أننا أجرينا تحليلاً مجمعاً لدراستين أظهر تساوي احتمالية الإصابة بنقص الصفائح الدموية لدى الحالات الخطيرة من البالغين (نسبة الأرجحية : 1.19, 95% مجال الثقة : 0.51-2.77). مما يدل على أن احتمالية الإصابة بنقص الصفائح الدموية لدى مرضى ملاريا المتصورة النشيطة يمكن أن تتباين بين الأطفال والبالغين حسب الحالة المناعية للمريض. كما أشارت إحدى الدراسات إلى ارتفاع معدلات البولة ومصل البيليروبين لدى المرضى المصابين بملاريا المتصورة النشيطة والنقص الحاد في الصفائح الدموية عن المرضى المصابين بالنقص الطفيف في الصفائح الدموية أو الذين لا يعانون منه أصلاً (القيمة الاحتمالية <0.001 في جميع أوجه المقارنة). وأظهر تحليل مجمع لدراستين أخرتين نسبة مماثلة من نوبات النزف مع نقص في الصفائح الدموية لدى مرضى المتصورة النشيطة الحادة والملاريا المنجلية الحادة (القيمة الاحتمالية =0.09). مما عنى أن كلاً من عدوى ملاريا المتصورة النشيطة و الملاريا المنجلية من الممكن أن تتواجد مع نوبات النزف، في حال حدوث تغير في عدد الصفائح الدموية لدى المرضى المصابين بالعدوى. وأظهر تحليل مجمع لدراستين أخرتين تساوي احتمالية الوفاة من جراء النقص الحاد في الصفائح الدموية متساوية بين مرضى ملاريا المتصورة النشيطة والملاريا المنجلية (نسبة الأرجحية : 1.16, 95% مجال الثقة : 0.3-4.6). مع ذلك، نظراً لمحدودية الدراسات وصغر حجم العينات ضمن الدراسات الفرعية التي قدمت معلومات سريرية ذات صلة، فإن ثقتنا في التقديرات المذكورة محدودة

الاستنتاجات: قدمت المراجعة القائمة بعض الأدلة على وجود علاقة سريرية بين النقص الحاد في الصفائح الدموية وملاريا المتصورة النشيطة. ولإثبات هذه النتائج، هناك حاجة إلى إجراء دراسات مصممة جيداً وواسعة النطاق في المستقبل تشمل المرضى المصابين بعدوى ملاريا المتصورة النشيطة من بلدان مختلفة ومن حالات وبائية أخرى من مختلف الفئات العمرية والجنسانية.

Translated from English version into Arabic by Heba Kandel, proofread by Mahmoud Sami, through

TRANSLATORS
WITHOUT BORDERS

间日疟与恶性疟患者相比较患严重血小板减少症的系统综述和荟萃分析

Cho Naing, Maxine A Whittaker

摘要

引言：间日疟原虫是人类疟原虫中地理分布最广泛的物种。免疫病理学研究表明，血小板是宿主对疟疾感染先天免疫应答的重要组成部分。本研究的目的是对间日疟患者的血小板减少症进行量化，并确定与恶性疟相比，间日疟患者严重血小板减少症的相关风险。

主要内容：本研究对间日疟患者血小板减少症的相关可用文献进行了系统综述和荟萃分析。在与卫生相关的电子数据库中搜索相关研究，遵循系统综述和荟萃分析指南的首选报告项目进行分析。本研究纳入了 58 项观察性研究(n = 29,664)。10.1%的间日疟患者观察到严重血小板减少症(< 50,000/mm^3)和非常严重血小板减少症(< 200,000/mm^3)。11 项观察性研究的荟萃分析显示，间日疟和恶性疟患者发生严重/非常严重的血小板减少的风险是相同的(*OR*: 1.98, 95% *CI*: 0.92-4.25)。这表明血小板减少症在间日疟和恶性疟中都是常见症状。一项研究表明，重症间日疟患儿罹患严重血小板减少症的风险较重症恶性疟患儿高(*OR*:2.8, 95% *CI*: 1.48-5.29)。然而，对两项研究的综合分析显示，间日疟和恶性疟成人重症病例出现血小板减少症的风险相当(*OR*: 1.19, 95% *CI*: 0.51-2.77)。这表明在间日疟中血小板减少症的风险可能因儿童和成人的免疫状况不同而产生差异。有一项研究报道，间日疟合并严重血小板减少症患者尿素和血清胆红素水平高于轻度血小板减少或无血小板减少症的患者(所有比较 P < 0.001)。另两项研究的综合分析显示，间日疟和恶性疟的重症患者的血小板减少症的发生率相似(P = 0.09)。这意味着间日疟原虫和恶性疟原虫感染均可能出现出血，感染者血小板计数也会发生变化。另两项研究的联合分析显示，间日疟和恶性疟患者的死亡率风险相当(*OR*: 1.16, 95% *CI*: 0.3-4.6)。由于在提供临床相关信息的研究中，小样本数量的研究较少，评估置信度有限。

结论：本综述为间日疟严重血小板减少症的临床相关性提供了一些证据。为了证实这些发现，我们需要在不同国家、不同年龄和性别群体中，对感染间日疟原虫的患者进行精心设计大规模的前瞻性研究。

Translated from English version into Chinese by Translated by Xin-Yu Feng, edited by Pin Yang

CHINESE CENTER FOR DISEASE CONTROL AND PREVENTION
NATIONAL INSTITUTE OF PARASITIC DISEASES

Thrombopénie sévère dans le paludisme à *P. vivax* et *P. falciparum* : revue systématique et méta-analyse

Cho Naing, Maxine A Whittaker

Résumé

Contexte: *Plasmodium vivax* est le parasite du paludisme humain dont l'aire de répartition géographique est la plus vaste. Des études immunopathologiques ont mis en évidence l'importance du rôle des plaquettes (thrombocytes) dans la réponse immunitaire innée de l'hôte à ces parasites. L'objectif de notre étude était de quantifier la thrombopénie parmi les patients infestés par *P. vivax* et de déterminer les risques associés de thrombopénie sévère, par rapport au paludisme à *P. falciparum*.

Discussion: Nous avons entrepris une revue systématique et une méta-analyse de la littérature existante à propos de la thrombopénie dans le paludisme à *P. vivax*. Nous avons recherché les études pertinentes dans les bases de données électroniques médicales. Nous avons suivi les recommandations PRISMA relatives aux critères et items à rapporter. Nous avons inclus 58 études observationnelles (*n* = 29 664) dans notre revue. Une thrombopénie grave (< 50 000/mm^3) à très grave (< 20 000/mm^3) a été observée chez 10,1 % des patients infestés par *P. vivax*. Une méta-analyse de 11 études observationnelles a montré un risque égal de développer une thrombopénie sévère ou très sévère entre les patients infectés par *P. vivax* et par *P. falciparum* (*OR* : 1,98, IC à 95 % de 0,92 à 4,25). Cela indique que la thrombopénie est une manifestation aussi fréquente des deux formes de paludisme. Une étude a montré un risque de thrombopénie très sévère plus élevé parmi les enfants souffrant de paludisme sévère à *P. vivax* que parmi ceux infestés par *P. falciparum* (*OR* : 2,8, IC à 95 % de 1,48 à 5,29). Une analyse regroupée de deux études a toutefois donné un risque égal parmi des cas sévères adultes (*OR* : 1,19, IC à 95 % de 0,51 à 2,77). Cela indique que le risque de développer une thrombopénie au cours d'un paludisme à *P. vivax* peut varier selon le statut immunitaire, aussi bien chez les enfants que chez les adultes. Une étude a rapporté un taux plus élevé d'urée et de bilirubine sérique chez les patients présentant un paludisme à *P. vivax* et une thrombopénie sévère que chez ceux qui présentaient une thrombopénie moins grave ou une numération thrombocytaire normale ($P < 0{,}001$ dans toutes les comparaisons). Une analyse regroupée de deux autres études a révélé une proportion similaire d'épisodes hémorragiques avec thrombopénie chez les patients souffrant d'un paludisme sévère à *P. vivax* et à *P. falciparum* ($P = 0{,}09$). Cela implique qu'une infestation par *P. vivax* aussi bien que par *P. falciparum* peut s'accompagner d'épisodes hémorragiques si la numération thrombocytaire des patients diminue. Une analyse regroupée de deux autres études a donné un risque égal de mortalité en cas de thrombopénie sévère dans le paludisme à *P. vivax* et à *P. falciparum* (*OR* : 1,16, IC à 95 % de 0,3 à 4,6). En raison du petit nombre d'études et de la petite taille des échantillons dans le sous-ensemble d'études qui ont fourni des informations cliniquement pertinentes, notre confiance dans ces estimations est limitée.

Conclusion: La présente revue a fourni quelques données probantes sur l'importance clinique de la thrombopénie sévère dans le paludisme à *P. vivax*. Afin de confirmer ces observations, nous avons besoin de grandes études prospectives bien conçues, menées auprès de patients infestés par *P. vivax* dans différents pays, dans d'autres contextes épidémiologiques et dans différents groupes d'âge et de sexe.

Translated from English version into French by Suzanne Assenat, proofread by matoudiallo, through

TRANSLATORS
WITHOUT BORDERS

Тяжёлая форма тромбоцитопении у больных доброкачественной трёхдневной малярией в сравнении с молниеносной трёхдневной (тропической) малярией: систематический обзор и метаанализ

Чоу Найн, Максин А Уиттакер

Аннотация

Аннотация. *Plasmodium vivax* является географически наиболее широко распространённым видом малярийных плазмодиев человека. Иммунопатологические исследования показали, что тромбоциты представляют собой важную составляющую врождённой иммунной реакции организма против заражения малярией. В настоящем исследовании ставилась цель количественного выражения тромбоцитопении у больных доброкачественной трёхдневной малярией, а также определение сопутствующих рисков возникновения тяжёлой формы тромбоцитопении у указанной группы пациентов в сравнении с молниеносной трёхдневной (тропической) малярией.

Описание исследования. Мы проделали систематический обзор и метаанализ имеющейся литературы по тромбоцитопении в случаях доброкачественной трёхдневной малярии. По электронным базам данных медицинской информации был выполнен поиск исследований, имеющих отношение к изучаемому вопросу. Данный обзор выполнялся в соответствии с рекомендациями по предпочтительным параметрам отчётности для систематических обзоров и метаанализа. В настоящий обзор вошли пятьдесят восемь обсервационных исследований (n = 29 664). Тромбоцитопения от тяжёлой формы (<50 000/мм^3) до крайне тяжёлой (<20 000/мм^3) наблюдалась у 10,1% пациентов, заражённых возбудителем *доброкачественной трёхдневной малярии.* Метаанализ 11 обсервационных наблюдений продемонстрировал равный риск развития тяжёлой/крайне тяжёлой форм тромбоцитопении как среди больных доброкачественной трёхдневной малярией, так и у пациентов, заражённых тропической малярией (*OR*: 1,98; 95% *CI*: 0,92-4,25). Следовательно, проявление тромбоцитопении в равной степени широко распространено как в случаях доброкачественной трёхдневной, так и тропической малярии. Исследование указало на повышенный риск тромбоцитопении у детей с крайне тяжёлой формой доброкачественной трёхдневной малярии, в сравнении с теми, кто страдает тяжёлой формой тропической малярии (*OR*: 2,8; 95% *CI*: 1,48-5,29). Однако при проведении совокупного анализа двух исследований было выявлено, что среди взрослых с тяжёлой формой заболевания также присутствует равный риск (*OR*: 1,19; 95% *CI*: 0,51-2,77). Таким образом, можно утверждать, что риск развития тромбоцитопении при доброкачественной трёхдневной малярии может колебаться в зависимости от иммунного статуса как у детей, так и у взрослых. По данным одного исследования, у пациентов с доброкачественной трёхдневной малярией и тяжёлой формой тромбоцитопении наблюдался повышенный уровень мочевины и билирубина крови в сравнении с теми, у кого не было тромбоцитопении или присутствовала лёгкая форма данного заболевания (P<0,001 во всех сравнениях). Сводный анализ двух других исследований продемонстрировал сходную пропорцию случаев кровотечения при тромбоцитопении у пациентов с тяжёлой формой доброкачественной трёхдневной малярии и у тяжёлых больных тропической малярией (P=0,09). Это указывает на то, что заражение возбудителем *доброкачественной трёхдневной малярии* и *тропической малярии* может сопровождаться кровотечениями, при условии изменения количества тромбоцитов у заражённых пациентов. Сводный анализ двух других исследований выявил одинаковый риск смертности при тяжёлой форме тромбоцитопении у пациентов как с доброкачественной

трёхдневной, так и с тропической малярией (*OR*: 1,16; 95% *CI*: 0,3-4,6). Наша уверенность в оценках носит ограниченный характер вследствие небольшого числа исследований с малыми размерами выборок в подгруппе исследований, результаты которых содержат представляющую для нас интерес клиническую информацию.

Заключение. Настоящий обзор предоставил некоторое обоснование клинической значимости тяжелой формы тромбоцитопении у пациентов с доброкачественной трёхдневной малярией. Для подтверждения выводов требуются хорошо спланированные крупномасштабные перспективные исследования среди больных, заражённых *возбудителем доброкачественной трёхдневной малярии,* которые следует проводить в разных странах и в различных эпидемиологических условиях среди групп, различных по возрасту и полу.

Translated from English version into Russian by Liudmila Tomanek, proofread by Natalia Potashnik, through

Trombocitopenia grave en pacientes con malaria por vivax comparada con malaria por falciparum: revisión sistemática y metaanálisis.

Cho Naing, Maxine A. Whittaker

Resumen

Antecedentes: *Plasmodium vivax* es la especie geográficamente más extendida entre los parásitos de malaria en humanos. Estudios inmunopatólogicos demostraron que las plaquetas son un componente importante de la respuesta inmune innata del huésped contra las infecciones de malaria. Los objetivos de este estudio eran cuantificar la trombocitopenia en pacientes con malaria por vivax y determinar los riesgos asociados de una trombocitopenia grave en pacientes con malaria por vivax comparada con malaria por falciparum .

Parte principal: Emprendimos una revisión sistemática y metaanálisis de la literatura disponible sobre trombocitopenia en malaria por vivax. Investigamos estudios relevantes en bases de datos electrónicas relacionados con la salud. Seguimos la directiva de los Ítems de referencia para publicar revisiones sistemáticas y metaanálisis . Cincuenta y ocho estudios observacionales (n = 29 664) se incluyeron en la revisión actual. Trombocitopenia grave (<50 000/mm^3) a trombocitopenia muy grave (<20 000/mm^3) se observó en 10,1% de pacientes con infección por *P. vivax*. Un metaanálisis de 11 estudios observacionales demostraron igual riesgo de desarrollar trombocitopenia grave o muy grave entre los pacientes con malaria por vivax y aquellos con malaria por falciparum (*O*: 1,98, 95% *IC*: 0,92-4,25). Esto indica que la trombocitopenia se manifiesta tan comúnmente en malaria por vivax como en malaria por falciparum. Un estudio reveló un riesgo mayor de desarrollar trombocitopenia muy grave en niños con malaria grave por vivax que con malaria grave por falciparum (*O*: 2,8, 95% *IC*: 1,48-5,29). Sin embargo, un análisis combinado de dos estudios demostró igual riesgo entre casos graves en

adultos (*O*: 1,19, 95% *IC*: 0,51-2,77). Esto indica que el riesgo de desarrollar trombocitopenia en malaria por vivax puede variar según el estado inmune tanto en niños como adultos. Un estudio informó niveles más altos de urea y de bilirrubina sérica en pacientes con malaria por vivax y trombocitopenia grave que entre aquellos con trombocitopenia leve o sin trombocitopenia (P<0,001 en todas las comparaciones). Un análisis combinado de otros dos estudios mostró una proporción similar de episodios de sangrado con trombocitopenia en pacientes con vivax grave y en pacientes con falciparum grave (P=0,09). Esto implicó que ambas infecciones, por *P. vivax* y por *P. falciparum* podían presentarse con episodios de sangrado, si había un cambio en el recuento de plaquetas en los pacientes infectados. Un análisis combinado de otros dos estudios reveló un riesgo igual de mortalidad por trombocitopenia tanto en pacientes con malaria por vivax y con malaria por falciparum*O*: 1,16, 95% *IC*: 0,3-4,6). Nuestra confianza en las estimaciones es limitada debido a un número bajo de estudios con tamaños de muestra pequeños dentro del subconjunto de estudios que brindaban información clínicamente relevante.

Conclusión: la revisión actual dio cierta prueba de la relevancia clínica de la trombocitopenia grave en la malaria por vivax. Para corroborar estos hallazgos, se necesitan estudios prospectivos a gran escala y bien diseñados entre pacientes infectados con *P. vivax* de países diferentes y de otros escenarios epidemiológicos entre grupos de edades y géneros diversos.

Translated from English version into Spanish by Lidia Lariño, proofread by soledadlescano, through

TRANSLATORS
WITHOUT BORDERS
