# Supplementary material for: Severe thrombocytopaenia in patients with vivax malaria compared to falciparum malaria: a systematic review and meta-analysis
Source: Infect Dis Poverty. 2018 Feb 9;7:10. doi: 10.1186/s40249-018-0392-9 (PMC5808388; doi:10.1186/s40249-018-0392-9)
Supplement: Supplementary file 3 — The excluded studies (DOC 37 kb) [file 40249_2018_392_MOESM3_ESM.doc]

Additional file 2 The excluded studies

| Reference. | Reason |
| --- | --- |
| 7 | case study |
| 72 | imported malaria |
| 73 | not possible to extract data |
| 74 | no data on vivax |
| 75 | no comparison with *P.falciparum* |
| 76 | pregnant women |
| 77 | case study |
| 78 | pregnant women |
| 79 | imported malaria |
| 80 | case report |
| 81 | diagnostic study |
| 82 | less than 10 cases |
| 83 | polymorphism study |
| 84 | study on cellular particles |
| 85 | case study |
| 86 | biomarkers |
| 87 | case study |
| 88 | case series |
| 89 | imported malaria |
