# Supplementary material for: Severe thrombocytopaenia in patients with vivax malaria compared to falciparum malaria: a systematic review and meta-analysis
Source: Infect Dis Poverty. 2018 Feb 9;7:10. doi: 10.1186/s40249-018-0392-9 (PMC5808388; doi:10.1186/s40249-018-0392-9)
Supplement: Supplementary file 4 — Characteristics of the included studies (DOC 119 kb) [file 40249_2018_392_MOESM4_ESM.doc]

Additional file 3. Characteristics of the included studies

| Reference. | Country | Setting | Design | Study period | Male | Mean age ±SD or (range) | Total /vivax cases | Diagnosis | Other malaria | TCP  (x10^3^/μl) | Remarks |
| --- | --- | --- | --- | --- | --- | --- | --- | --- | --- | --- | --- |
| 11 | Indonesia | hosp | pros | 4/ 2004-12/2012 | 53.2^g^ | >15 yr 60.3% | 66421/19552^λ^ | TBF/RDT | *Pf*, mix | mean,  ML, MD, S | UV vs UF;  vs non- malaria; mortality |
| 12 | India | hosp | pros | 11/2008-12/2010 | 70% | 79% :<10Y | 676/380 | TBF/RDT ,PCR | *Pf,* mix | mean, ML,MD, S | - |
| 16 | Pakistan | OPD | CS | 7/2011-9/2011 | 67.3% | 30.2± 14.6 | 107/107 | TBF /RDT | *Pv* only | ML,MD, S,VS | - |
| 17 | Brazil | clinic | pros | 3/2006-10/2006 | 69.7% | (18-60) | 86/86 | TBF & PCR | *Pv* only | ML | - |
| 18 | Colombia | OPD | CS | 2011-2013 | 59.3% | 19md  (12-33) | 1328/673 | TBF & PCR  (10%) | *Pf,* mix | mean | passive surveillance |
| 19 | India | hosp | pros | 2011-2013 | NA | (1-60) | 360/231 | TBF/  RDT | *Pf,* mix | not defined | no cutoff level for TCP grades |
| 20 | Malaysia | hosp | pros | 9/ 010-10/2011 | 77% | 24  (18-42) | 295/43 | TBF & PCR | *Pf, Pk* | ML | other malaria ,SV;UV |
| 21 | Brazil | hosp | pros | NA | 82.9% | 41.8 ±13.6 | 35/35 | TBF & PCR | *Pv* only | ML | TCP vs non-TCP; |
| 22 | India | hosp | retro | 1/2011-  12/2011 | 68% ^g^ | (15-85) | 100/54 | TBF | *Pf*, mix | ML, S | UV vs UF |
| 23 | Turkey | hosp | CC | NA | NA | (14-37) | 110/60 | TBF | *Pv* only | mean | not under Rx(50 healthy control) |
| 24 | Thailand | clinic | pros | 5/2001-6/2001 | 71.2% | (20-70 ) | 2149/646 | TBF | *Pf*, mix | mean,  ML | other malaria |
| 25 | India | hosp | retro | 3/2010-4/2010 | NA | 42  (18-66) | 30/30 | TBF | *Pv* only | VS | VS: 16x10^3^;  ^2 deaths with bleeding^ |
| 26 | Brazil | hosp | Retro  CC | 12/2011-11/2013 | 72.5% | 39^md^  (26-49) | 80/80 | TBF & PCR | *Pv* only | ML,MD, S |  |
| 27 | Brazil | clinic | CS | 3/2004 –  4/2004* | 62.4% | 30  (21-43) | 127/85 | PCR | *Pf*, mix | mean | UV vs UF vs mix |
| 28 | India | hosp | retro | 1/2012-12/2012 | 70% | 5.2 ±3.5 | 79/47 | RDT | *Pf* | ML | UV/Uf |
| 29 | India | hosp | pros | 7/2013-  10/2013 | 75% | <18 | 19/12 | TBF & PCR | *Pv* only | S | UV;12:SV  non-infected (n=  50), non-severe (n12) severe (n=12 |
| 30 | Pakistan | hosp | pros | 2/2007-  12/ 2011 | 75% | 33.2 ± 8.3 | 824/160 | TBF | *Pf*, mix | ML, MD, S | UV vs UF vs mix |
| 31 | India | clinic | pros | NA | 58.5% | 37.4 ±14.2 | 1563/973 | TBF | Pf | mean,  ML  S | UV vs UF vs mix |
| 32 | India | hosp | pros | 6/2008-12/2008 | 75% | ≤12 yr | 38/35 | TBF | *Pf,* mix | ML | SV |
| 33 | Pakistan | hosp | pros | 11/2008-  11/2010 | NA | adults | 121/39 | TBF | *Pf* | ML, MD, S | Uv vs Uf |
| 34 | India & Saudi Arabia | hosp | pros | 2012-2013 | NA | mostly children | 197/106 | TBF/  RDT | *Pf* | ML |  |
| 35 | Korea | hosp | CC | 4/2000-8/2006 | 81.8% | 26.1 ±11.1  (24-60) | 141/55 | TBF | *Pv* only | mean,  ML. MD | 86:non-malaria /healthy control |
| 36 | India | hosp | pros | 9/2003-12/2005 | 72.5%** | 29.6± 11.7 | 1091/456 | PCR | *Pf,Pv* | mean,MD | UV, SV (n=40) |
| 37 | India | hosp | pros | 8/2007-11/2008 | 67% | children | 150/65  303/103 | PCR | *Pf*, mix | MD | SV vs UV  SV150/303 |
| 38 | India | hosp | pros | 1/2007-7/2008 | NA | adults | 1064/460 | PCR^b^ | *Pf,* mix | ML, MD, S |  |
| 39 | India | hosp | pros | 6/2011-12/2011 | 70% | (1-60) | 546 | PCR | *Pv* only | mean,  ML | outpatients |
| 40 | India | hosp | pros | 1/2007-12/2008 | 60.2% | Adults | 539/221 | PCR | *Pf,* mix |  | SV vs Sf |
| 41 | Thailand | hosp | CS | 1/2009-12/2009 | 59.5% | 23  (16-36) | 703/351 | TBF | *Pf* | mean,  risk estimate | UV vs UF;  vs non-malaria |
| 42 | Korea | hosp | retro | 1/2005-12/2009 | 71.2% | 42.5 ± 14.7 | 352/352 | TBF | *Pv* only | ML, MD, S, VS | - |
| 43 | Brazil | clinic | CS | 2008-2013 | 78% | 37.7 ± 14.7 | 186/186 | TBF | *Pv* only | mean,  ML, MD, S | UV |
| 44 | India | hosp | retro | 8/2009-10/2009 | NA | NA | 680/338 | TBF/RDT | *Pf,* mix | MD  <100K 68% | under antimalarial Rx |
| 45 | Sudan | hosp | pros | 9/2009-12/2011 | 55.6% | 4.9± 2.4 | 79/18 | TBF | *Pf* | ML MD | SV |
| ~~46~~ | PNG | hosp, | pros | 10/2006-12/2009 | 55.6% | 33(26-61) M;  0.5-10 yr | 340/27 | PCR^d^ | *Pf*, mix | mean | SV vs Sf |
| 47 | Colombia | clinic,  hosp | retro | 1997-2007; 2005-2010 | 62.9% | 25.9± 16.6 | 862/311 | TBF | *Pf*, mix | mean,  ML,MD  S,VS | mean age of 610 out of total 862 pts |
| 48 | Pakistan | clinic | CS | 1/2009-12/2009 | 81% | 40.5+17.3 | 97/97 | TBF /RCT | *Pv* only | ML,S |  |
| 49 | India | hosp | CC | NA | NA | 4-56 | 25/9 | NA | *Pf,* | mean | healthy controls |
| 50 | India | hosp | pros | NA | 66.7% | 28.4 ±7.4 | 100/24 | TBF | *Pf* | mean, <150 | UV vs UF |
| 51 | India | hosp | pros | NA | 68.3% | 36.8 ±11.2 | 1188/300 | TBF | *Pf*, mix | ML | 240:UV; 60:SV; 888pt bleed |
| 52 | India | hosp | pros | 6/2010-1/2011 | 71.9% | NA | 711/488 | TBF/RDT | *Pf* | ML |  |
| 53 | India | hosp | retro | 1/2010-  12/2010 | 80.8% | 33.17 ±14.85 | 213/213 | TBF | *Pv* only | ML MD |  |
| 54 | Korea | hosp | retro | 1/1996-12/1999 | 92% | 23^md^  (17-77) | 101/101 | TBF | *Pv* only | ML, MD, S |  |
| 55 | Indonesia | hosp | pros | 4/2004-4/2008 | 54.6% | 53.7%: >9/12-m | 181/102 <3mo;  1560/668 infants | TBF | *Pf*, mix | mean; MD, S | UV vs UF vs mix |
| 56 | India | 3^0^ care, hosp | pros | 1/2013-6/2014 | 66.15% | >16;  mainly 21-30 | 100/65 | TBF/RDT | *Pf* | ML, MD, S |  |
| 57 | Pakistan | hosp | pros | 9/2006-12/2006 | 100% | 28.2± 7  (12-60) | 502/100 | TBF | *Pf,* mix | Mean  ML | pregnancy excluded |
| 58 | Pakistan | hosp | CC | 1/2009-12/2011 | NA | 40^md^  (30-45.2) | 282/182 | PCR | *Pv* only | mean, ML,MD, S | plasma samples;  100 UV; 82:SV;100: healthy |
| 59 | India | hosp | retro | 1/2009-12/2011 | 59.7%= | 31.11±13.14 | 172/172 | TBF/RDT | *Pv* only | not defined | SV,  8 spontaneous bleedin |
| 60 | Brazil | clinic | retro | NA | 74.5% | 28(22-38) | 71/47 | TBF | *Pf*, | ML | UV vs UF |
| 61 | India | hosp | pros | 6/2011-3/2012 | NA | adults | 900/900 | TBF/RDT | *Pv* only | MLMD,  S,VS | 200:SV |
| 62 | India | hosp | pros | 8/2004-7/2006 | 86% | 30 (all adults) | 131/60 | QBC | *Pf* | mean, ML, MD, S | SV vs UV |
| 63 | India | hosp | retro | 6/2009-9/2009 | 56% | 5.4 ± 3.7 | 232/108 | TBF/RDT | *Pf*, mix | ML,S | S:39% of SV |
| 64 | Pakistan | hosp | CS | 2009 & 2010 | 52.3% | NA | 64/64  176/128 | TBF, PCR | *Pf* | S |  |
| 65 | India | hosp | pros | 12/2010-11/2012 | 68.8% | 12md;  (0-18) | 85/61 | TBF/RDT | *Pf* | ML, S | UV vs UF |
| 66 | India | hosp | CC | 1yr | 58% | 0-17:  (38% U5) | 100/45 | TBF/RDT | *Pf*, mix | ML, MD, S | UV vs UF |
| 67 | Korea | hosp | retro | 7/1998-9/2001 | 75% | 35 ±16.7 | 44/44 | TBF | *Pv* only | ML, MD | TCP 75% |
| 68 | India | hosp | retro | 4/2008-8/2008 | 60% | >18 | 50/74 | TBF/RDT | *Pf,* mix | ML | SV |
| 69 | Indonesia | hosp | RCT | before 2008 | 90% | adults | 162/63 | TBF | *Pf* | S | At day 0,  day 28 |
| 70 | Pakistan | hosp | CS | 10/2003-8/2005 | 60% | 39.31+7.63 | 162/85 | TBF/  RDT | *Pf* | ML, MD, S | UV vs UF |
| 71 | India | hosp | pros | 6 mth | 92.7% | (10-70) | 150/150 | TBF | *Pv* only | ML,MD,S |  |

a: % of total vivax malaria; b: for patients with TCP ; c: per µl; d: for population-based study; e: vivax malaria for the year 2009; f: PCR confirmed vivax malaria; g: based on total malaria cases; *from another source;**for severe malaria; ***for uncomplicated group; ^λ^ : based on inpatients & outpatients events; b/f-after: before and after treatment of malaria; CC: case-control; CS: cross-sectional study; Eth: ethinicity ;G1: grade 1; HC: health centre; hos: hospital based study; h/o travel: history of recent travelling to malaria endemic country; hypo: hypoendemic; mean: mean platelet counts; MD: moderate thrombocytopaenia ; ML : mild thrombocytopaenia; mth: months;national: nationality; nurs’: nursing home; OPD: outpatient department; Pk:*Plasmodium Knowlesi*; pop: population; pros: prospective study, Retro: retrospective study; 3^0^ care: tertiary care center; S: severe thrombocytopaenia; SV: severe vivax malaria; TBF thick & thin blood film; U5: under 5 year old; UF:uncomplicated falciparum malaria; UV: uncomplicated vivax malaria; VS: very severe.
