# Supplementary material for: Severe thrombocytopaenia in patients with vivax malaria compared to falciparum malaria: a systematic review and meta-analysis
Source: Infect Dis Poverty. 2018 Feb 9;7:10. doi: 10.1186/s40249-018-0392-9 (PMC5808388; doi:10.1186/s40249-018-0392-9)
Supplement: Supplementary file 5 — Proportions of mild and moderate thrombocytopaenia in vivax malaria (DOC 62 kb) [file 40249_2018_392_MOESM5_ESM.doc]

Additional file 4. Proportions of mild and moderate thrompocytopaenia in vivax malaria

| Reference | Country | Patients  with thrombocytopaenia | Total | Proportion % |
| --- | --- | --- | --- | --- |
| Mild thrombocytopaenia |  |  |  |  |
| 17 | Brazil | 62 | 86 | 72.1 |
| 19 | India | 108 | 231 | 46.8 |
| 21 | Brazil | 22 | 35 | 62.9 |
| 22 | India | 3 | 54 | 5.6 |
| 24 | Thailand | 377 | 646 | 58.4 |
| 27 | Brazil | 17 | 43 | 39.5 |
| 28 | India | 17 | 47 | 36.2 |
| 31 | India | 632 | 973 | 65.0 |
| 34 | India | 48 | 106 | 45.3 |
| 37 | India | 9 | 103 | 8.7 |
| 39 | India | 468 | 546 | 85.7 |
| 40 | India | 91 | 221 | 41.2 |
| 43 | Brazil | 31 | 186 | 16.7 |
| 48 | Pakistan | 88 | 97 | 90.7 |
| 49 | India | 4 | 9 | 44.4 |
| 51 | India | 11 | 60 | 18.3 |
| 52 | India | 89 | 488 | 18.2 |
| 56 | India | 3 | 65 | 4.6 |
| 57 | Pakistan | 100 | 502 | 19.9 |
| 58 | Pakistan | 10 | 182 | 5.5 |
| 59 | India | 35 | 62 | 56.5 |
| 61 | India | 80 | 200 | 40.0 |
| 68 | India | 41 | 50 | 82.0 |
| 70 | Pakistan | 24 | 85 | 28.2 |
| Moderate thrombocytopaenia |  |  |  |  |
| 36 | India | 9 | 40 | 22.5 |
| 56 | India | 30 | 65 | 46.2 |
| 58 | Pakistan | 93 | 182 | 51.1 |
