# Supplementary material for: Severe thrombocytopaenia in patients with vivax malaria compared to falciparum malaria: a systematic review and meta-analysis
Source: Infect Dis Poverty. 2018 Feb 9;7:10. doi: 10.1186/s40249-018-0392-9 (PMC5808388; doi:10.1186/s40249-018-0392-9)
Supplement: Supplementary file 6 — Distribution of mean platelet counts (DOC 29 kb) [file 40249_2018_392_MOESM6_ESM.doc]

Additional File 5. Distribution of mean platelet counts

| Study | Vivax malaria | | | Healthy controls | | | *p* value |
| --- | --- | --- | --- | --- | --- | --- | --- |
|  | mean | standard deviation | total | mean | standard deviation | total |  |
| Erel, 2001[23] | 128.2 | 61.46 | 60 | 259.6 | 69.2 | 50 | < 0.0001 |
| Kim, 2008[35]* | 91.1 | 47 | 55 | 249.9 | 43.4 | 52 | < 0.0001 |
| Kotepui,  2014[41] | 89 | 17.5 | 351 | 242 | 34 | 4282 | < 0.0001 |
| Raza, 2014 [58] | 32.17 | 11.19 | 79 | 175 | 45.78 | 100 | < 0.0001 |

*Transformed from median and interquartile range with the Hozo formula [14]
