# Supplementary material for: Severe thrombocytopaenia in patients with vivax malaria compared to falciparum malaria: a systematic review and meta-analysis
Source: Infect Dis Poverty. 2018 Feb 9;7:10. doi: 10.1186/s40249-018-0392-9 (PMC5808388; doi:10.1186/s40249-018-0392-9)
Supplement: Supplementary file 7 — Proportion of bleeding manifestation in patients with vivax malaria (DOC 32 kb) [file 40249_2018_392_MOESM7_ESM.doc]

Additional file 6. Proportion of bleeding manifestation in patients with vivax malaria

| Study | Cases with bleeding episodes | Total cases | Proportion % | 95%CI lower limit | 95%CI upper limit |
| --- | --- | --- | --- | --- | --- |
| Tanwar, 2012 [12] | 63 | 380 | 16.8 | 13.2 | 20.6 |
| Singh, 2011  [63] | 3 | 23 | 13.0 | 4.5 | 32.1 |
| Kaushik,2012 [32] | 1 | 17 | 5.9 | 1.05 | 27 |
| Rizvi,2011  [59] | 8 | 35 | 22.9 | 12.1 | 39.0 |
| Pooled proportion % |  |  | 15 | 9 | 21 |
