# Supplementary material for: Severe thrombocytopaenia in patients with vivax malaria compared to falciparum malaria: a systematic review and meta-analysis
Source: Infect Dis Poverty. 2018 Feb 9;7:10. doi: 10.1186/s40249-018-0392-9 (PMC5808388; doi:10.1186/s40249-018-0392-9)

Additional File 7. Comparison of mortality with severe thrombocytopaenia between severe vivax malaria and severe falciparum malaria

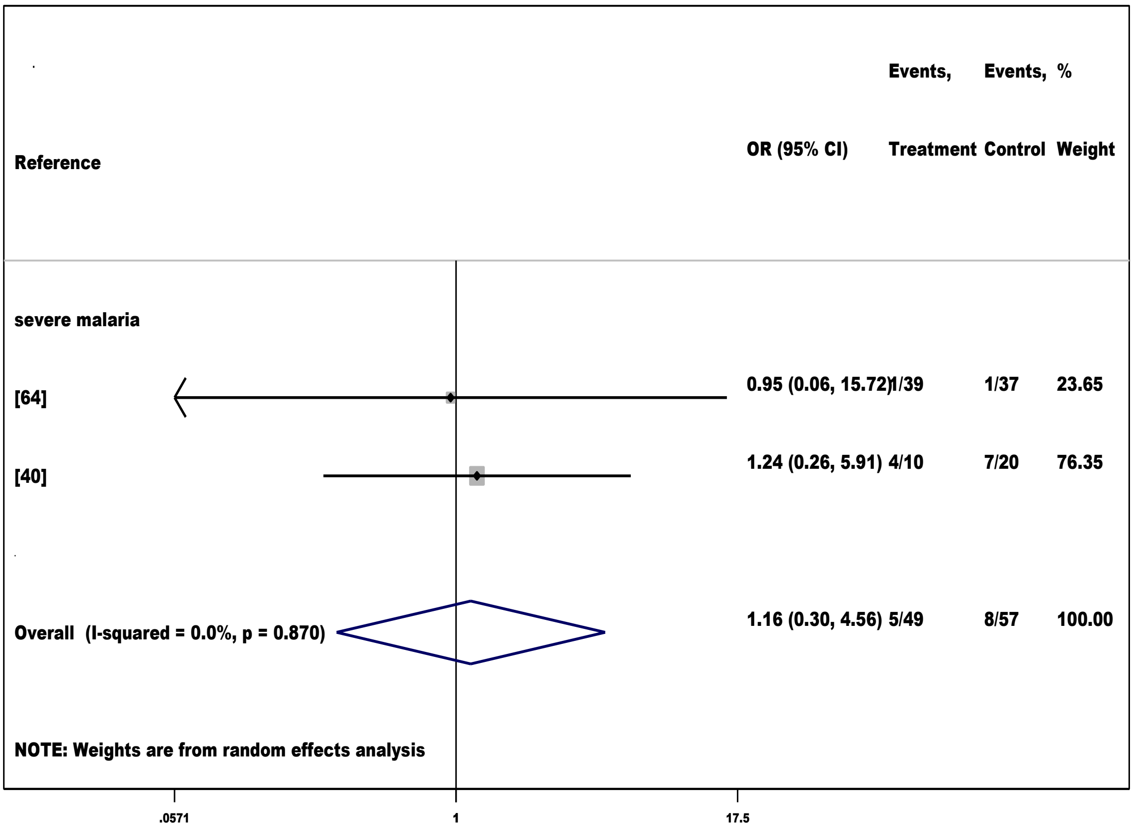

Supplement: Supplementary file 8 — Comparison of mortality with severe thrombocytopaenia between severe vivax malaria and severe falciparum malaria (PDF 99 kb) [file 40249_2018_392_MOESM8_ESM.pdf]
